# Field-Testing and Refinement of the Organisational Health Literacy Responsiveness Self-Assessment (Org-HLR) Tool and Process

**DOI:** 10.3390/ijerph17031000

**Published:** 2020-02-05

**Authors:** Anita Trezona, Sarity Dodson, Emma Fitzsimon, Anthony D. LaMontagne, Richard H. Osborne

**Affiliations:** 1 Faculty of Health, Deakin University, Burwood, VIC 3125, Australia; sdodson@hollows.org (S.D.); tony.lamontagne@deakin.edu.au (A.D.L.); 2 The Fred Hollows Foundation, Carlton, VIC 3053, Australia; 3 Inner North West Primary Care Partnership, Brunswick, VIC 3056, Australia; EmmaFi@inwpcp.org.au; 4 Centre for Global Health and Equity, Faculty of Health, Arts and Design, Swinburne University of Technology, Hawthorn, VIC 3122, Australia; rosborne@swin.edu.au

**Keywords:** health literacy, health literacy responsiveness, health systems, access, health service improvement, self-assessment, Org-HLR self-assessment tool

## Abstract

Health literacy refers to the skills and knowledge that influence a person’s ability to access, understand and use information to make health-related decisions, which are influenced by the complexity of their health needs and the demands health services place on them. The aim of this study was to field-test the Organisational Health Literacy Responsiveness (Org-HLR) tool and process to determine their utility in assessing health literacy responsiveness and for supporting organisations to plan health literacy-related improvement activities. Four organisations in Victoria, Australia, field-tested the Org-HLR tool. Data were collected through direct observation, participant feedback, and focus groups. Forty-three individuals participated in field-testing activities, and 20 took part in focus group meetings. Themes relating to the applicability and utility of the Org-HLR self-assessment tool and process were identified. Field-testing resulted in a number of refinements to the tool and process. Twenty-eight indicators were removed, 29 were rephrased to improve their clarity, and four new indicators were added. The revised Org-HLR self-assessment tool contains six dimensions, 22 sub-dimensions and 110 performance indicators. The Org-HLR tool and process were perceived as useful for assessing health literacy responsiveness, prioritising improvement activities, and establishing a benchmark for monitoring and evaluation of improvements over time. Testing generated an improved Org-HLR tool and assessment process that are likely to have utility across a broad range of health and social service sector organisations.

## 1. Introduction

Health literacy has been defined as “the cognitive and social skills which determine the motivation and ability of individuals to gain access to, understand and use information in ways which promote and maintain good health” [1]. People with low functional health literacy may have less knowledge about their health conditions and treatments, poorer overall health status, and higher rates of hospitalisation than the general population [2,3,4,5]. Low functional health literacy may also impact an individual’s ability to participate in decision-making, follow care recommendations, implement health-promoting behaviours, and engage with preventative health services [6,7,8].

The health literacy skills and abilities that are required by individuals to effectively interact with health services are likely to depend on the complexity of those services and the demands they place on people [9,10]. Health systems are complex, and health organisations may be structured and operate in ways that make it difficult for people to access and engage with information and care. The interaction between an individual’s health literacy capabilities and the complexity of health systems is now widely acknowledged, and public health professionals, researchers and policy makers are increasingly advocating that organisations increase their responsiveness through system, process and practice improvements [10,11,12,13].

Health literacy responsiveness has been defined as “the provision of services, programs and information in ways that promote equitable access and engagement, that meet the diverse health literacy needs and preferences of individuals, families and communities, and that support people to participate in decisions regarding their health and social wellbeing” [14]. Trezona et.al described the characteristics of a health literacy responsive organisation as including a culture that promotes equity and inclusiveness, effective leadership and management, robust data collection, monitoring and communications systems and processes, effective communication practices, a commitment to building workforce capability, and a commitment to engaging meaningfully with the communities they serve, as well as other health and social service sector organisations [14].

Improving health literacy responsiveness is concerned with improving the functions and performance of health and social care organisations to ensure they deliver effective, high quality, person-centred services and programs. Organisational self-assessments can be useful for improving performance and effectiveness by supporting benchmarking and monitoring, guiding continuous quality improvement activities, and promoting organisational learning [15]. They are increasingly being utilised to guide organisational ‘diagnosis’ and needs identification processes, as well as to facilitate goal setting and quality improvement planning [16,17,18].

The Organisational Health Literacy Responsiveness (Org-HLR) tool and assessment process were developed to support organisations to assess their health literacy responsiveness and to prioritise and plan quality improvement activities [19]. The Org-HLR tool is divided into three parts: (i) reflection, (ii) self-rating, and (iii) priority-setting. The associated assessment process involves a series of cross-team or multidisciplinary workshops. An initial reflection workshop encourages group discussion about health literacy concepts, the specific health literacy needs of clients and communities, and the role of organisations in responding to these needs. A second self-rating workshop enables organisations to rate their health literacy responsiveness against a set of assessment criteria and performance indicators. The final priority-setting workshop supports organisations to prioritise their improvement activities based on the weaknesses they identify in the self-rating workshop.

We developed the Org-HLR tool and assessment process to address the limited availability of health literacy responsiveness self-assessment tools, as well as limitations with the content and administration format of existing tools. The aim of this study was to field-test the Org-HLR self-assessment tool and process to determine their utility in supporting organisations to assess health literacy responsiveness and plan health literacy-related improvement activities. Specifically, the study sought to: (1) determine the applicability and comprehensibility of the tool content (assessment dimensions, sub-dimensions and performance indicators); (2) identify the key strengths, limitations and benefits of the tool and process; and (3) identify the improvements required to enhance the utility and effectiveness of the tool and process for future users.

## 2. Materials and Methods

This study involved implementing the Org-HLR tool across four disparate health and social service sector organisations in Victoria, Australia, during which data were collected using direct observation, participant feedback, and focus group meetings. These data were used to determine the utility of the Org-HLR tool and assessment process and to identify areas for improvement.

### 2.1. Study Sites and Participants

Expressions of interest to participate were sought from health and social service sector organisations based in the north and west metropolitan regions of Melbourne, Australia. An email invitation was sent to the member organisations (*N* = 40) of a metropolitan Primary Care Partnership. Primary Care Partnerships are voluntary alliances of health and human service organisations that work together to improve access to, and coordination of services [20]. The invitation contained information on the aims, objectives and activities of the study, as well as an application form to participate. To be eligible, organisations had to meet three criteria: (i) relevance—demonstrated alignment of the study with existing health literacy priorities; (ii) capacity—staff availability to undertake the assessment process within the defined timeframes and a dedicated staff member to coordinate study activities in consultation with the research team; and (iii) authorisation—participation in the study authorised by a senior manager. Four organisations submitted an expression of interest and were selected to participate in the study (See Table 1). A total of 43 individuals, including managers, clinical staff, community development workers, health promotion practitioners, and administrative staff, participated in field-testing activities.

### 2.2. Procedure and Materials

Initial project meetings were held with participating sites in June 2016 to plan the field-testing activities. The sites were introduced to the Org-HLR self-assessment tool and process, and the steps and time commitment involved in undertaking the assessment were explained. The site teams were provided with an opportunity to ask questions about the Org-HLR self-assessment tool and process, and each site negotiated an approach for implementing the assessment process at their site.

The self-assessment workshops were conducted between July and October 2016. Workshops—in which discussions, decisions and ratings were recorded by using the Org-HLR self-assessment tool templates—were prepared and facilitated by the research team. The research team also developed assessment reports for each site to inform their future planning and evaluation activities.

### 2.3. Data Collection

Data were collected through direct observation and field notes, participant feedback (verbal and email), and focus group meetings. These data were organised according to the following categories: (i) assessment process; (ii) tool content; (iii) rating system; (iv) terminology and language; and (v) general comments. Specific feedback that was provided by participants during and between workshops was recorded in a separate log, which was also categorised as above. A log of the content issues that were identified by the research team was also maintained.

Four focus group meetings (one per site) were conducted at the end of the field-testing period. The meetings involved a structured discussion on participants’ perceptions of the Org-HLR self-assessment tool and process:

1. How applicable and comprehensible was the content of the Org-HLR tool?
2. What were the benefits of undertaking the assessment?
3. What were the key strengths of the Org-HLR tool and assessment process?
4. What were the key limitations of the Org-HLR tool and assessment process?
5. How can the Org-HLR tool and assessment process be improved?

All focus group meetings were conducted over 45–60 min. They were audio recorded and transcribed verbatim. The transcripts were provided to participants to confirm the accuracy of the data. The data were then consolidated so that only the substantive information from the discussions was retained.

### 2.4. Data Analysis

A general inductive approach to data analysis was applied [21]. The analysis was guided by the research aims, and themes were derived through an inductive coding process. The data were then cleaned, condensed, collated, and categorised into the high order themes (codes) that were identified in the raw data by the lead researcher. A second round of analysis was undertaken to identify a second order of themes for the coding framework. Two other researchers then independently tested the coding framework and the accuracy of the coding process by examining portions of the raw data against the codes. After confirmation, the final coding framework (comprised of 12 high order categories and 58 codes) was applied to the whole data set again in a third and final round of analysis, through which the most prominent and relevant themes relating to the applicability and utility of the Org-HLR self-assessment tool and process were identified.

### 2.5. Ethics Approval

This study was approved by the Deakin University Human Research Ethics Committee (Study ID: 2012-295). Written informed consent was obtained from a manager at each organisation to participate in the field-testing activities, as well as from individuals who participated in the focus group meetings.

## 3. Results

### 3.1. Initial Orientation to the Org-HLR Assessment Process and Selected Approach across Participating Sites

While the Org-HLR self-assessment process was expected to be implemented in three parts—(i) reflection activity, (ii) self-rating activity, (iii) priority-setting activity—in order to meet the specific needs of some organisations, minor modifications were necessary. For example, some sites already had a health literacy action plan in place, so they did not perceive a need to undertake the priority-setting activity. The approach to the assessment for each site is described in Table 2. Providing this flexibility allowed us to test the adaptability of the Org-HLR self-assessment tool and process during the field-testing period. Importantly, all sites completed the self-rating activity and were therefore exposed to all Org-HLR tool assessment dimensions and performance indicators. All four sites opted not to use the “*external policy and funding environment dimension*,” as they deemed this to be outside their sphere of influence.

### 3.2. Applicability of the Org-HLR Tool Content

Overall, participants perceived the assessment dimensions, sub-dimensions and performance indicators of the Org-HLR tool as comprehensive, meaningful and appropriate. Participants reported that there were no specific gaps in the assessment dimensions, sub-dimensions or performance indicators. They reported that all content areas were relevant to the concept of health literacy responsiveness. However, they suggested that it would be more useful to incorporate the “*external policy and funding environment*” domain into the reflection component of the self-assessment process.

Some performance indicators were either not well understood by participants or required clarification, and some items appeared to be repetitive. While some items had been deemed to contain important distinctions during the development of the Org-HLR tool, these distinctions were not obvious to participants during the assessment workshops. Examples of items that were not well understood or required clarification included ‘*Staff are encouraged and supported to accurately document/record the number and type of services provided*’ and ‘*Health literacy is viewed as an individual and community asset and right.*’ The assessment dimension “*undertaking data collection and community needs identification*” was an area perceived to have repetitive items, in that it contained three items related to assessing access barriers. These issues were addressed in the refinement process, which is described later.

### 3.3. Strengths of the Org-HLR Tool and Assessment Process

Field-testing revealed a number of strengths of the Org-HLR tool and assessment process (Table 3). Strengths included that they were informed by empirical research, developed in the Australian context, comprehensive, appropriate in breadth and scope, and structured into logical and appropriate assessment dimensions and sub-dimensions. With regard to the assessment process, participants reported two key strengths: (i) the facilitated workshop format and (ii) the ability to collect quantitative and qualitative information. These strengths resulted in the reported benefits of undertaking the assessment process, described below.

### 3.4. Benefits of Undertaking the Org-HLR Assessment Process

Participants in the field-testing activities reported that the Org-HLR assessment process provided a number of benefits at both the individual staff member and organisational levels (Table 4). These included informing strategic and operational planning processes and establishing a baseline of organisational performance, both of which enabled monitoring and evaluation. The process also provided an opportunity for knowledge exchange between individuals within an organisation, as well as professional development.

### 3.5. Limitations of the Org-HLR Tool and Assessment Process

Field-testing also highlighted a number of limitations (Table 5) that informed the refinements made to the tool and process as part of this study. The first limitation was related to the term health literacy itself. Some participants reported that their organisation avoided using the term, as it was not understood by staff and was considered jargon. Participants reported that the tool was too long and repetitive in some assessment dimensions. Similarly, some participants reported that the length of time required to complete the assessment process may not be feasible for some organisations and would exclude some staff from participating, particularly clinical staff.

The most commonly reported limitation of the tool was the rating system and criteria applied in both the self-rating and priority setting activities. Participants reported these to be overly complicated and confusing, and, in some cases, the descriptors did not apply well to the performance indicators. Other limitations reported included the potential duplication of other self-assessment processes (such as cultural competence and diversity) and an inability to judge performance in areas that did not relate to the participants’ roles within the organisation (for example, clinical staff perceived they could not make judgements about how their organisation makes financial decisions).

### 3.6. Refinements to the Org-HLR Tool and Assessment Process

The field-testing informed a number of refinements to the Org-HLR tool and assessment process. The first of these was related to the self-rating component of the Org-HLR tool. Of the 135 original performance indicators, 29 were rephrased to improve their clarity, 25 were removed, and four new indicators were added. Performance indicators were removed if they duplicated or overlapped with others, were too vague to have sufficient meaning for users, or were deemed irrelevant. The first assessment dimension, “*external policy and funding environment*,” was removed from the self-rating tool and incorporated into the reflection tool. The sub-dimensions “*providing supportive working environments*” and “*providing practice tools and resources*” were merged into one sub-dimension, as both relate to providing support for staff. All assessment dimension and sub-dimension headings were reframed as action-orientated statements, as participants perceived one-word headings to be vague. For example, the assessment dimension “*workforce*” was rephrased to “*recruiting, supporting and developing the workforce*.” A detailed description of each assessment dimension was also added to better orientate users to the meaning and intended focus of the assessment dimensions. The revised Org-HLR self-assessment tool contained six dimensions, 22 sub-dimensions and 110 performance indicators, as shown in Table 6.

To address the reported difficulty with the rating system, the global rating system was removed and the template was modified to allow for a rating against each performance indicator. The five-point scale was retained, but a ‘not applicable’ option was incorporated, and the descriptors for the rating levels were simplified. Table 7 shows the original and revised rating scales and descriptions.

Based on participant feedback regarding the complexity of the priority-setting tool, the rating system and criteria were revised by incorporating three components that were designed to support organisations to prioritise and plan their improvement activities: (i) the level of importance (reflects the level of impact this has on the organisation’s performance); (ii) the level of urgency; and (iii) the resources required (an assessment of whether additional human or financial resources are required). The rating criteria are shown in the priority-setting tool template that is provided in Supplementary File 1.

Finally, more detailed instructions were incorporated in the user guide to increase the capacity of organisations to undertake the self-assessment process. This included more specific guidance on establishing the assessment team, what to expect when undertaking the assessment process, the role of the facilitator, and how to prepare for workshops and complete the self-assessment reports. The templates provided with the Org-HLR tool were also refined to better support data collection and the reporting of self-assessment results.

## 4. Discussion

In this study, we field-tested and refined the Org-HLR self-assessment tool and process across four organisations, which provided substantial evidence that they were comprehensive, useful, generated valuable data, and have the potential to initiate change in organisations such that they become more responsive to the health literacy needs of community members. Specifically, from the perspective of participating sites, the Org-HLR tool and process were useful for identifying organisational strengths and weaknesses in health literacy responsiveness, as well as for prioritising improvement activities. In addition, it was useful for informing organisational level strategic and operational planning processes, as well as program level and team-based planning. It was also useful for benchmarking organisational performance relating to health literacy responsiveness, and will therefore enable the monitoring and evaluation of performance and improvements over time.

While the tool and process were developed to support organisational diagnosis and need assessment, they are likely to provide additional benefits related to organisational learning and professional development. Participants consistently reported that participating in the assessment process facilitated cross-team knowledge exchange and collaboration, and it increased their knowledge and understanding of health literacy and health literacy responsiveness.

Specific recommendations for improvements by participants were incorporated into an improved version of the Org-HLR tool and process. Three key improvements to enhance the utility of the tool were reported to be (i) the removal of the “external policy and funding environment” assessment dimension from the self-rating component of the tool (and instead incorporating it into the reflection activity), (ii) simplifying the rating systems and criteria, and iii) developing more detailed instructions within the Org-HLR user guide.

While participants did not perceive the “external policy and funding environment” assessment dimension to be relevant for self-assessment, they acknowledged it to be a key enabler of organisational health literacy responsiveness, confirming the views of participants who were involved in the development of the Org-HLR [14]. Incorporating a discussion on this into the reflection activity maintains an emphasis on the need for organisations to be supported by and be aware of the policy and funding environment. However, this places the discussion within the broader context of organisational readiness, as well as potential drivers of organisational practice and performance.

A key strength of our approach to field-testing the Org-HLR was the immersion of the research team in the assessment. This immersion enabled the direct observation of the strengths and limitations of the Org-HLR tool and process, the collection of feedback in real time, and continuous quality improvements to the tool and process throughout the field-testing period. This was particularly useful for determining the applicability and comprehensibility of the tool content, as the specific terms and concepts that were difficult for participants to understand or that were not relevant became readily apparent.

However, having the research team, namely the tool developer, facilitate the assessment process for all participating sites was also a limitation of this study, as it removed the opportunity to test the usability of the Org-HLR tool and assessment process with novice users. In order to be effective, self-assessment tools must be usable without the involvement of external facilitators or experts [22]. We expect that the revised version of the Org-HLR tool and process will be feasible to administer by novice users, as many of the issues described by participants have been addressed, and a detailed user-guide has been developed. A second limitation is that our study design did not incorporate a cost–benefit analysis, so we were unable to quantify the average cost to organisations of completing the Org-HLR assessment. Other limitations of this study include the small number of sites that were involved in field-testing activities and the limited number of organisation/sector types that were represented. For these reasons, further evaluations of the utility of the Org-HLR tool and process across a broader range of settings and without external support would be beneficial. Future evaluations should also seek to assess the cost–benefit of the Org-HLR assessment process.

### Considerations for Future Users

A number of factors are likely to enhance the successful implementation of the assessment process by future users. Firstly, an adequate level of organisational readiness to undertake the self-assessment process is essential, as is commitment to implementing system, process and practice improvements. This is critical to ensuring that once the intense period of initial engagement is over, there is ongoing momentum and commitment to implementing agreed actions. An important issue that emerged through this study was the variability in acceptance and use of the term health literacy, as well as the varying ways in which health literacy is defined and understood. By undertaking organisational awareness-raising activities about health literacy definitions and concepts prior to completing the self-assessment process, staff will be better equipped to efficiently judge the extent to which organisations demonstrate health literacy responsiveness across the broad range of assessment dimensions and performance areas described in the Org-HLR tool. This should include a discussion about the range of health literacy-related concepts such as consumer participation, person-centred care, cultural competence, access, equity and diversity [23,24,25,26,27], all of which are described in the Org-HLR user guide (available from the authors).

Organisations will need to ensure that they allocate adequate time and resources to the assessment process in order to produce meaningful results that can guide improvement activities. The assessment process will be enhanced by ensuring that a broad range of staff, including managers, practitioners, administration, and quality assurance and facilities staff, are involved in the assessment workshops, as the collective knowledge of the assessment group allows all assessment dimensions to be confidently judged. Finally, as with any organisational improvement initiative, careful consideration should be afforded to selecting an appropriate facilitator, as this person plays a critical role in preparing and managing the assessment process, including the development of assessment reports. The facilitator should be selected on the basis that they have the skills to undertake these activities, as well as the ability to provide a safe and inclusive environment in which all staff have the opportunity to contribute to the discussions. A knowledge of health literacy, health literacy responsiveness, and related concepts may also be an advantage.

Finally, the Org-HLR tool and process may be utilised to complement or enhance organisational accreditation processes and other related self-assessment processes such as cultural competence, gender equity and diversity assessments. This can be achieved by identifying and focusing on the assessment dimensions, sub-dimensions and performance indicators that build on, rather than duplicate, existing processes.

## 5. Conclusions

The Org-HLR self-assessment tool and process were developed through robust, participatory processes to guide the development of health literacy responsiveness in the Australian context. While field-testing generated several improvements, the tool and process were found to have utility in assessing health literacy responsiveness and planning improvement activities. The revised version of the Org-HLR tool is now available for use by organisations wanting to improve their health literacy responsiveness. We expect that it will have utility across a broad range of health and social service sector organisations, including community health, hospitals, women’s health, primary care, local governments, and other peak bodies and not-for-profit organisations; however, further testing and tailoring of the tool and process in other settings are warranted.

## Figures and Tables

**Table 1:** Description of study sites.

Site	Number of Service Locations	Number of Staff *	Number of Service Users	Types of Services Delivered
Site #1 Large community health service	>40	>850	>110,000 service users	Medical services
				Dental services
				Allied health services
				Mental health services
				Aged and disability services
				Refugee health services
				Counselling services
				Specialist health services
				Health promotion
				Chronic disease programs
Site #2 Large public hospital	2	>6500	>85,000 admissions per year	Acute medical services
				Specialist medical services
				Surgical services
				Rehabilitation services
				Aged care services
				Outpatient services
				Community programs
Site #3 Medium community health service	4	>240	>5900 service users	Allied health
				Dental services
				Medical services
				Counselling services
				Alcohol and drug services
				Youth services
				Child and family services
				Aged and disability services
				Refugee health services
				Health promotion
				Chronic disease programs
Site #4 State wide Not for Profit (social service)	1	110	Approximately 5000 client interactions per year.	Advisory Line
				Counselling
				Education Services
				Respite Programs (Aged, Disability, Mental Health, Older Families and Young Carers)
				Policy and Research
				Support Groups
				Workplace Training and Solutions

* Includes volunteers.

**Table 2:** Details of the Organisational Health Literacy Responsiveness (Org-HLR) assessment process implemented at each site.

Site	Rationale for Participating	Approach to Assessment	Participants
Site #1 Large community health service	To establish a baseline of current organisational practice and performance. A follow-up assessment to be completed in two years to determine progress.	Health literacy action plan already in place—opted not to undertake the reflection and priority setting activities. Two self-rating workshops (2 h each) were delivered. The first covered assessment dimensions 4, 5 and 6. The second covered assessment dimensions 2, 3 and 7.	A group of practitioners (*N* = 9) from various teams participated in the first workshop. A group of managers/senior managers (*N* = 3) participated in the second workshop.
Site #2 Large public hospital	To establish a baseline of current organisational practice and performance, as well as to identify and prioritise actions for implementation.	Due to time constraints, the organisation opted not to undertake the reflection and priority setting activities. Two self-rating workshops (1–2 h each) were delivered.	Practitioners and managers (*N* = 11) from various disciplines from the medical department participated in the workshops.
Site #3 Medium community health service	To identify gaps in health literacy work undertaken to date, identify and prioritise new actions for the future, and engage staff from across a wider range of teams in the planning and implementation of health literacy activities.	Implemented the Org-HLR process in full. A combined reflection and self-rating workshop (4 h) and a priority setting workshop (2 h) were delivered.	Practitioners and managers (*N* = 13) from various teams across the organisation participated in the whole process.
Site #4 State wide not for profit (social service)	To increase staff awareness of health literacy and to increase their engagement in health literacy activities.	Implemented the Org-HLR process in full. Two self-rating workshops (3 h each) and a priority-setting workshop (2 h) were delivered. The first self-rating workshop incorporated reflection activity.	Practitioners and staff (*N* = 7) from various teams across the organisation participated in the whole process.

**Table 3:** Perceived strengths of the Org-HLR tool and process.

Strength	Description	Example Quote
Evidence-based	The Org-HLR tool was informed by empirical research and developed in the Australian context.	“Staff value and appreciate the evidence base that the work has come from.”
Appropriate scope and breadth	The scope and breadth of the Org-HLR tool is comprehensive and appropriate for assessing whole of organisation health literacy responsiveness.	“It needs to be broad… If you’re looking at a whole of organisation approach to something, you do have to have a broad assessment.”
Logically structured	The Org-HLR tool is logically and appropriately structured into relevant assessment dimensions and sub-dimensions.	“The way it has been broken down into the different domains of leadership and culture, and workforce… I found that really helpful… it is good to break it down into those subsections, otherwise it can be overwhelming.”
Facilitated workshop format (conversation-based).	The workshops format encourages participation from a broad range of people, which enables cross-team conversations, collaboration, team building and knowledge exchange.	“It was good to have people from different parts of the organisation… Having that diversity (of staff representation) is really useful…” “There was something different about this process. What I liked about this process that was different was the conversational component… There was that thing of really hearing (other) experiences.”
Generates both quantitative and qualitative data	The use of a quantitative rating system supports the identification of strengths and limitations, as well as the benchmarking and monitoring of improvements over time. The qualitative component supports the documentation of examples that may inform planning.	“I think the item level (rating) is important because it can drive some of that conversation around what our weaknesses and strengths are.” “The other thing I really like about the rating is that idea of being able to go back and do it again and see change.” “Examples are good. Getting people to think about, reflect on examples and jot them down, and sharing that is useful.”

**Table 4:** Perceived benefits of undertaking the Org-HLR assessment process.

Benefit	Description	Example Quote
Supports organisational planning processes	The process informed or will inform organisational planning processes, including strategic plans, operational plans, and specific health literacy action plans.	“(To support our) new strategic planning process, working out where the health literacy work and plan sits, who is responsible I think this process is making that clearer for us.”
Supports evaluation and monitoring	The process was useful for establishing a baseline of organisational performance in health literacy responsiveness, and this will be used to monitor and evaluate improvements over time.	“The primary purpose was to provide a kind of baseline assessment, and a method for ongoing assessment… and to understand whether we have achieved the objectives of our health literacy plan.”
Enables cross-team collaboration	The process provided an opportunity for and encouraged cross-team discussions and collaboration on health literacy responsiveness.	“Having some forums where there is cross-team discussions is the only way we break down silos, and I think that’s one of the great benefits of this exercise.” “Giving them the opportunity to be a part of (this process) is quite meaningful in itself. Hopefully it gives people a sense that this is something that they’re contributing to, that they are a part of.”
Promotes knowledge exchange	The process enabled participants to share their perspectives on organisational performance, including examples of good practices and current challenges across their disciplines/work areas.	“It’s good to have other people’s perspectives because senior managers have a broader view of what’s happening, but they might not actually have the knowledge of what happens in practice.”
Promotes awareness and understanding of health literacy	The process increased staff awareness and understanding of health literacy and health literacy responsiveness, including strategies they could implement to address/improve them.	“People appreciated being able to come together and talk about health literacy and get a better understanding of what it means.”
Promotes reflection and learning opportunity	The process encouraged participants to reflect on their own practice and the practices of their organisation. They also reported that the process provided them with an opportunity to learn about health literacy responsiveness and to learn more about their organisation.	“I think that absolutely will make it easier for staff to realise it’s not just about words, it’s about how I behave, the spaces we have, the systems and processes (in place).” “It does raise your curiosity though, reading the different dimensions. For me I thought if I don’t know about it should I be finding out about it.”

**Table 5:** Perceived limitations of the Org-HLR tool and process.

Limitation	Description	Example Quote
Terminology	The term health literacy is not used by some organisations, as it is not well understood by all staff and/or they perceive it to be jargon and abstract.	“One of the limitations of the words health literacy is that it very much points to literacy, to words and language, and I think that is its biggest handicap as a notion, as a concept.” “We don’t use the terminology health literacy, so everyone’s got a slightly different take on it or they take it very literally as literacy—reading and writing skills, rather than thinking broader than that.”
Length of the tool	The Org-HLR tool was too long and repetitive in some assessment dimensions	“I did find it a bit drawn out.” “I’d like to see it simplified… from a usability point of view I tend to think shorter is better.”
Global rating system and criteria of self-rating tool	The global rating system was perceived as confusing, complicated, and as not allowing for an accurate assessment of each performance indicator. The rating criteria were also perceived as overly complicated and as not applying well against some assessment areas.	“I think it is easier for each statement to have a rating rather than just the overall (sub-dimension) rating.” “Make that a bit clearer around how to rate.”
Criteria of priority setting tool	The rating criteria for the priority-setting tool were perceived as complicated.	“We did also talk about the priority setting tool rating system being two pronged—importance versus urgency.”
Duplication with other self-assessment tools and processes	Participants reported that this Org-HLR tool and process overlapped with other self-assessment tools and quality improvement processes (e.g., cultural competence and accreditation). This is may lead to the duplication of effort and action plans.	“Another issue is the overlap with existing accreditation (processes) and existing evaluation tools, and the fact that we’ve already been through this process and evaluated a whole stack of things.” “The risk there is, if we have action plans coming out of a number of different self-assessments that are looking at the same thing, we end up having different people approaching the same problem in different ways.”
Time required	Some participants perceived the time required to complete the assessment to be prohibitive. As a result, some staff would not be able to participate (i.e., clinical staff) and it would be difficult to ensure consistent representation throughout the assessment process.	“At the beginning (of planning the assessment) I thought the time commitment was going to be a really hard ask.”
Staff roles and representation	Some participants perceived that parts of the tool were not relevant to their role or work area; therefore, they could not make an informed judgement about organisational performance in that area.	“It assumes, and this is why it’s important to have representation from across the organisation, that we know as individuals what’s going on (in other parts of the organisation) and we just don’t.” “I think for it to work here, chunking (breaking sections down) by who was responsible and their work group, rather than health literacy titles might make it easier to get it done.”

**Table 6:** Revised Org-HLR tool assessment dimensions, sub-dimensions and number of performance indicators.

Assessment Dimensions	Sub-Dimensions	Original Indicators	Revised Indicators
1. Supportive leadership and culture	1.1. Allocates financial resources	3	3
	1.2. Demonstrates leadership and commitment	4	5
	1.3. Makes health literacy an organisational priority	4	3
	1.4. Promotes equity and diversity	4	4
	1.5. Fosters a person-centred philosophy	3	3
2. Supportive systems, processes and policies	2.1. Undertakes data collection and community needs identification	9	7
	2.2. Undertakes performance monitoring and evaluation	5	5
	2.3. Undertakes service planning and quality improvement	7	5
	2.4. Ensures effective communication systems and processes are in place	8	8
	2.5. Ensures written internal policies and procedures are in place	6	6
3. Supporting access to services and programs	3.1. Provides and appropriate service environment	3	3
	3.2. Supports initial entry and ongoing access to services and programs	8	8
	3.3. Provides outreach services	3	3
4. Community engagement and partnerships	4.1. Undertakes community consultation and enables consumer participation	8	6
	4.2. Works in partnership with other organisations	6	5
5. Communication practices and standards	5.1. Applies communication principles and standards	10	8
	5.2. Provides health information effectively	6	5
	5.3. Uses media and technology effectively	5	4
	5.4. Provides health education programs	3	3
6. Recruiting, supporting and developing the workforce	6.1. Recruits an appropriate workforce	4	3
	6.2. Provides supportive working environments, practice tools and resources	3 + 8 *	5
	6.3. Provides ongoing professional development	11	8

* Originally two separate sub-dimensions.

**Table 7:** Rating scale and descriptions of the original and revised Org-HLR tool.

Original Rating	Original Rating Description	Revised Rating	Revised Rating Description
1	There is no evidence that this occurs, and there is no support/commitment internally for undertaking work in this area.	0	Not at all
2	There is no evidence that this occurs, but the organisation has made a commitment to it and planning has commenced.	1	Minimally
3	There is evidence that this occurs sporadically across some parts of the organisation, but it is undertaken inconsistently and significant improvements are required.	2	Partially
4	There is evidence that this occurs consistently across most parts of the organisation, but improvements are required to embed it into organisational systems and processes.	3	Substantially
5	This is routine practice that is consistently undertaken across all areas of the organisation and has been embedded into organisational systems and processes.	4	Fully
		N/A	Not applicable

## Supplementary Materials

The following are available online at https://www.mdpi.com/1660-4601/17/3/1000/s1, File S1: Org-HLR Rating Criteria.

## References

[1] Nutbeam D. (1998). Health Promotion Glossary, World Health Organization. http://www.who.int/healthpromotion/about/HPR%20Glossary%201998.pdf?ua=1.

[2] Herndon J.B., Chaney M., Carden D. (2011). Health literacy and emergency department out-comes: A systematic review. Ann. Emerg. Med..

[3] Wolf M.S., Gazmararian J.A., Baker D.W. (2005). Health literacy and functional health status among older adults. Arch. Intern. Med..

[4] Tokuda Y., Doba N., Butler J.P., Paasche-Orlow M.K. (2009). Health literacy and physical and psychological wellbeing in Japanese adults. Patient Educ. Couns..

[5] Jessup R.L., Osborne R.H., Beauchamp A., Bourne A., Buchbinder R. (2017). Health literacy of recently hospitalised patients: A cross-sectional survey using the Health Literacy Questionnaire (HLQ). BMC Health Serv. Res..

[6] Institute of Medicine Health Literacy: A Prescription to End Confusion, Institute of Medicine, retrieved May 23 2014. http://www.nationalacademies.org/hmd/Reports/2004/Health-Literacy-A-Prescription-to-End-Confusion.aspx.

[7] Ishikawa H., Yano E. (2008). Patient health literacy and participation in the health-care process. Health Expect..

[8] Van der Heide I., Uiters E., Rademakers J., Struijs J.N., Schuit A.J., Baan C.A. (2014). Associations among health literacy, diabetes knowledge, and self-management behavior in adults with diabetes: Results of a dutch cross-sectional study. J. Health Commun..

[9] Pleasant A., Cabe M.A., Martin L., Rikard R.V. (2013). A Prescription is Not Enough: Improving Public Health with Health Literacy.

[10] Koh H.K., Baur C., Brach C., Harris L.M., Rowden J.N. (2013). Toward a systems approach to health literacy research. J. Health Commun..

[11] DeWalt D., McNeill J. (2013). Integrating Health Literacy with Health Care Performance Measurement.

[12] Brach C., Keller D., Hernandez L.M., Baur C., Parker R., Dreyer B., Schyve P., Lemerise A., Schillinger D. (2012). Ten Attributes of Health Literate Health Care Organisations: Discussion Paper.

[13] World Health Organization Regional Office for Europe (2013). Health Literacy: The Solid Facts.

[14] Trezona A., Dodson S., Osborne R.H. (2017). Development of the Organisational Health Literacy Responsiveness (Org-HLR) Framework in Collaboration with Health and Social Services Professionals. BMC Health Serv. Res..

[15] Ritchie L., Dale B.G. (2000). Self-assessment using the business excellence model: A study of practice and process. Int. J. Prod. Econ..

[16] Miller R., Bedney B., Guenther-Grey C. (2003). Assessing Organizational Capacity to Deliver HIV Prevention Services Collaboratively: Tales from the Field. Health Educ. Behav..

[17] Ford M.W. (2001). Baldrige Assessment and Organizational Learning: The Need for Change Management. Qual. Manag. J..

[18] Fountain M. (1998). The target assessment model as an international standard for self-assessment. Total Qual. Manag..

[19] Trezona A., Dodson S., Osborne R.H. (2018). Development of the Organisational Health Literacy Responsiveness (Org-HLR) self-assessment tool and process. BMC Health Serv. Res..

[20] Department of Health and Human Services, (2015) Primary Care Partnerships, Department of Health and Human Services, retrieved 1 April 2017. https://www2.health.vic.gov.au/primary-and-community-health/primary-care/primary-care-partnerships.

[21] Thomas D.R. (2006). A General Inductive Approach for Analyzing Qualitative Evaluation Data. Am. J. Eval..

[22] Ford M.W., Evans J.R. Models for Organizational Self-Assessment. Business Horizons. 2002 2 January 2017. https://www.researchgate.net/publication/4884983_Models_for_Organizational_Self-Assessment?enrichId=rgreq-1f7a6e2f3443860e63c540825c5a4e74-XXX&enrichSource=Y292ZXJQYWdlOzQ4ODQ5ODM7QVM6MTg5MTg1MzE1MDU3NjY3QDE0MjIxMTY3MDI2MjQ%3D&el=1_x_2.

[23] Batterham R.W., Hawkins M., Collins P.A., Buchbinder R., Osborne R.H. (2016). Health literacy: Applying current concepts to improve health services and reduce health inequalities. Public Health.

[24] Alper J. (2016). Integrating Health Literacy, Cultural Competence and Language Access Services: Workshop Summary.

[25] Lie D., Carter-Pokras O., Braun B., Coleman C. (2012). What do health literacy and cultural competence have in common? Calling for a collaborative health professional pedagogy. J. Health Commun..

[26] Levesque J.F., Harris M.F., Russell G. (2013). Patient-centred access to health care: Conceptualising access at the interface of health systems and populations. Int. J. Health Equity Health.

[27] Australian Commission on Safety and Quality in Health Care (2014). Health Literacy: Taking Action to Improve Safety and Quality, Australian Commission on Safety and Quality in Health Care. http://www.safetyandquality.gov.au/wp-content/uploads/2014/08/Health-Literacy-Taking-action-to-improve-safety-and-quality.pdf.

