# Supplementary material for: Field-Testing and Refinement of the Organisational Health Literacy Responsiveness Self-Assessment (Org-HLR) Tool and Process
Source: Int J Environ Res Public Health. 2020 Feb 5;17(3):1000. doi: 10.3390/ijerph17031000 (PMC7037726; doi:10.3390/ijerph17031000)
Supplement: Supplementary file 1 [file ijerph-17-01000-s001.pdf]

How to use this Tool:

Step 1: Identify Actions

Determine and record the actions required to improve your performance under each assessment dimension. The information you documented during the self-rating part of the assessment will support you to determine these actions. Some of the actions you have identified may need to be broken down into a set of smaller actions in order to make the planning process more manageable. It is important to spend an adequate amount of time determining these actions before completing steps 2-4 of this process.

Step 2: Rate Importance Level

Determine the level of importance of each action you have identified, and assign a rating according to the following criteria:

1: Unimportant
2: Minor importance
3: Moderate importance
4: High importance
5: Essential

Step 3: Rate Urgency Level

Determine the level of urgency of each action, and assign a rating according to the following criteria:

High: Needs to be implemented within 3 months
Medium: Needs to be implemented within 6- 12 months
Low: Can be implemented at any time

Step 4: Determine Resources Required

Determine and indicate the resourcing required for implementing each action according to the following criteria:

E: Can be achieved with existing resources
N: Additional staff or financial resources are required

When completing this part of the assessment process, keep in mind that the information you provide does not commit your organisation to action at this stage. Being thorough with your identification of actions, and honest about their level of importance, urgency and resourcing require will enable more effective decision making during future planning activities.

Area One: Leadership and Culture

A health literacy responsive organisation has a philosophy and values that are inclusive, person-centred and equity driven. It also has leaders, managers and decisions makers who drive and support effective financial management, service planning, change management and continuous quality improvement. In this assessment area you will explore the extent to which the leadership and culture within your organisation supports health literacy responsiveness.

Actions required to improve our performance	Importance	Urgency	Resources Required
**Allocating financial resources**			
**Leadership and commitment**			
**Health literacy is an organisational priority**			
**Equity and diversity focused**			
**Person-centred philosophy**			
